# The effect of warming ropivacaine on ultrasound-guided subgluteal sciatic nerve block: a randomized controlled trial

**DOI:** 10.1186/s12871-023-02332-5

**Published:** 2023-11-13

**Authors:** Jiliang He, Yijun Ma, Nannan Zhou, Jingpin Xu, Weidong Wu, Jiajie Jiang, Fengjiang Zhang

**Affiliations:** 1 Department of Anesthesiology, The Second Affiliated Hospital, Zhejiang University School of Medicine, No. 88 Jiefang Rd, Hangzhou, 310009 Zhejiang China; 2 Department of Anesthesiology, Cixi People Hospital Medical Health Group (Cixi People Hospital), NO.999 The Second Ring of the South Road, Hushan Street, Cixi, 315300 Zhejiang China; 3 Department of Anesthesiology, Ningbo Medical Center Lihuili Hospital, NO.57 Xingning Rd, Ningbo, 315040 Zhejiang China

**Keywords:** Sciatic nerve block, Temperature, Local anesthetics, Warming

## Abstract

**Background:**

There is a long latent period for the sciatic nerve block before a satisfactory block is attained. Changes in the temperature of local anesthetics may influence the characters of the peripheral nerve block. This study was designed to evaluate the effect of warming ropivacaine on the ultrasound-guided subgluteal sciatic nerve block.

**Methods:**

Fifty-four patients for distal lower limbs surgery were randomly allocated into warming group (group W, *n* = 27) or room tempeture group (group R, *n* = 27) with the ultrasound-guided subgluteal sciatic nerve block. The group W received 30 ml of ropivacaine 0.5% at 30℃ and the group R received 30 ml of ropivacaine 0.5% at 23℃. The sensory and motor blockade were assessed every 2 min for 30 min after injection. The primary outcome was the onset time of limb sensory blockade.

**Results:**

The onset time of sensory blockade was shorter in group W than in group R (16 (16,18) min vs 22 (20,23) min, *p* < 0.001), and the onset time of motor blockade was also shorter in group W than in group R (22 (20,24) min vs 26 (24,28) min, *p* < 0.001). The onset time of sensory blockade for each nerve was shorter in group W than in group R (*p* < 0.001). No obvious differences for the duration of sensory and motor blockade and the patient satisfaction were discovered between both groups. No complications associated with nerve block were observed 2 days after surgery.

**Conclusions:**

Warming ropivacaine 0.5% to 30℃ accelerates the onset time of sensory and motor blockade in the ultrasound-guided subgluteal sciatic nerve block and it has no influence on the duration of sensory and motor blockade.

**Trial registration:**

The trial was registered on October 3, 2022 in the Chinese Clinical Trial Registry (https://www.chictr.org.cn/bin/project/edit?pid=181104), registration number ChiCTR2200064350 (03/10/2022).

## Background

The sciatic nerve block is frequently applied alone or in combination with other peripheral nerve blocks for orthopedic procedures involving the foot and lower limb [1], with the advantage of little influence on respiratory and circulatory systems. One of the main disadvantages of sciatic nerve block is a long latent period before achieving a satisfactory blockade, despite the use of the ultrasound equipment, which is related to the thick nerve structure made by multiple layers [2]. It should be noted that the prolonged latency significantly prolongs the waiting time for patients in the preoperative room and diminishes the clinical efficiency, which appears to influence many anaesthetists’ decision to perform the sciatic nerve block when considered the most appropriate technique for patients. Unfortunately, despite the application of various strategies [3–5], this problem is still unresolved effectively in clinical practice.

In the literature, few studies focused on the effect of warming local anesthetics on peripheral nerve block [6]. To our knowledge, they were only limited to brachial plexus block. Several previous studies reported that warming local anesthetics could improve the onset of brachial plexus block [7–9]. However, this finding was still controversial [10]. Therefore, this study was designed to investigate the effect of warming ropivacaine on the ultrasound-guided subgluteal sciatic nerve block. The choice of the subgluteal approach is highly desirable in our study, because it can offer numerous advantages, including ease of administration, high success rates and minimal discomfort to patients. Besides, it can provide a greater range of anesthesia or analgesia in comparison to the popliteal level [3]. The primary outcome was the onset time of limb sensory blockade. The secondary outcomes were the onset time of sensory blockade for each nerve, the onset time of motor blockade, the duration of sensory and motor blockade and the patient satisfaction.

## Methods

The protocol utilized in this study was granted approval by the ethics committee at Cixi People’s Hospital on July 24, 2022, and subsequently reviewed and registered at the Chinese Clinical Trials Registry (ChiCTR-TRC-0000054) on October 3, 2022. This study was conducted from October 2022 to February 2023 at Cixi People’s Hospital.

Fifty-four adult patients (18 to 70 yr), classified as American Society of Anesthesiologists (ASA) Physical Status Class I to II, who underwent distal lower extremity surgery, were estimated for eligibility to enter this study. Patients with obesity (BMI ≥ 35), mellitus diabetes, or a history of coagulopathy or neurological disorder were excluded from the study. Following the acquisition of signed informed consent forms, all patients were randomly allocated into warming group (group W, *n* = 27) or room temperature group (group R, *n* = 27) using a computer-generated random number table. Details of the allocated group were given on colored cards contained in sequentially numbered, opaque, sealed envelopes. The group W received 30 ml of ropivacaine 0.5% at 30°C and the group R received 30 ml of ropivacaine 0.5% at 23°C.

The solutions of ropivacaine 0.5% were prepared with a 1:1 mixture of ropivacaine 1% (Qilu Pharmaceutical, Jinan, China) and normal saline (China Otsuka Pharmaceutical Co.,Ltd., Tianjin, China). For group R, the extension tubes and 20-ml syringes with ropivacaine 0.5% were stored in the operating room set at 23°C before initiating the block. For group W, the extension tubes and 20-ml syringes with ropivacaine 0.5% were stored in an incubator (DHG9011A, Jinghong Laboratory Instrument Co.,Ltd., shanghai, China) set at 30°C for at least 2 h before initiating the block. The solutions were all prepared by a nurse who was not involved in the performance of the intraoperative management, or data collection and analysis.

All patients were routinely prohibited from drinking for 6 h and fasted for 8 h. They were monitored by standard noninvasive monitors (MX500, Philips, Boeblingen, Germany) with blood pressure (BP), heart rate (HR), electrocardiograph (ECG) and blood oxygen saturation (SpO_2_) in the operating room. Additionally, oxygen was administered via a facemask at 3L/min.

Patients were positioned laterally with the affected limb elevated and flexed at the hip and knee. A ultrasound device (MP80, Mindray, Shenzheng, China) with a low-frequency (5–2 MHz), curved-array ultrasound transducer (C5-1S) was used. The ultrasound transducer was performed on the skin in the subgluteal region, oriented parallel to a line between the ischial tubercle and greater trochanteric for sciatic nerve imaging. On this level, the sciatic nerve could be visualized on sonography as an oval or flattened hyperechoic nodule in a transverse plane. Subsequently, the longitudinal section image of the sciatic nerve could be obtained by rotating the transducer 90 degrees from its original position. The position of the sciatic nerve was confirmed by a combination of longitudinal and transverse scanning. The width, thickness and depth of the sciatic nerve were measured on ultrasonography.

A 80-mm, 22-gauge needle (TWLB, Kindly Medical Devices Co., Ltd., Zhejiang, China) was carefully advanced in parallel with the ultrasound beam towards the sciatic nerve until the needle tip was positioned adjacent to the nerve tissues on ultrasound imaging. Subsequently, 2–3 ml of saline solution was injected to confirm the correct placement of needle tip by observing a spread around the sciatic nerve. After confirmation of negative aspiration, 30 ml of ropivacaine 0.5% (23℃ or 30℃) was incrementally injected to spread along the dorsal aspect of the nerve tissue without changing the initial position of the needle tip, within less than 20 s. In order to avoid intraneural injection, all injections were administered using 20-mL syringes in incremental doses. Meanwhile, the ultrasound video recorded the injection process around the sciatic nerve for subsequent assessment of the occurrence of intraneural injection. All nerve blocks were performed by a single anesthesiologist proficient in ultrasound regional anesthetic techniques, who remained blinded to group assignment.

The sensory and motor blockade was evaluated every 2 min for 30 min after injection. The pinprick (22G) was used on three regions of the lower limb for the assessment of sensory blockade, including the dorsal aspect of the foot (common peroneal nerve, CPN), the plantar aspect of the foot (tibial nerve, TN) and the lateral cutaneous side of the lower leg (sural nerve, SN). Sensation to pinprick was classified as follows: 0 = normal sensation (no block); 1 = blunted sensation (analgesia); 2 = absence of sensation (anesthesia). It was considered complete when patients received complete loss of pinprick sensation (score = 2). The onset time of sensory blockade was defined as the time from the end of injection to complete blockade in three dermatomes. The degree of dorsal and plantar flexion in the foot and toes was assessed as an evaluation index for motor blockade. It was classified as follows: 0 = normal movement; 1 = decreased movement; 2 = absence of movement (complete motor block). It was considered complete if the patient showed paralysis with dorsal and plantar flexion of the foot and toes (score = 2). The onset time of motor blockade was defined as the time from the end of injection to completion of motor block. Block failure was defined that the completion of sensory and motor block was not achieved whithin 30 min after injection and these patients were excluded from analysis. The time for resolution of motor blockade and the first request for pain medication were documented on the first day after surgery. Additionally, the neural complications for patients such as sensory disability and/or muscle weakness in the lower limbs were assessed on the second day after surgery. All data was collected by an investigator blinded to randomization. A comprehensive review of all videos was conducted by another investigator who was blind to all the information and data of patients, including the group assignment.

Intraneural injection was defined that any segment of the nerve showed significant swelling in a transverse plane on ultrasonography during administration of local anesthetics [11]. Patients with a poor video image during injection were excluded. In order to mitigate the effect of drug temperature changes on experimental results, those with injection time exceeding 20 s were also excluded.

After achieving successful sensory and motor blockade, LMA general anesthesia was induced using propofol at a dosage of 2–3 mg/kg, followed by maintenance with sevoflurane (2.0%-2.5%) while ensuring spontaneous breathing of the patient. During the surgery, sufentanil citrate was administered intermittently with dosage adjustments based on the patient’s noninvasive blood pressure, respiratory rate, and heart rate.

### Sample size calculation

In advance, we conducted a preliminary experimental study with 10 patients in each group. The results indicated that the mean onset time of sensory block was 19.8 ± 2.27 min in warming group and 17.8 ± 1.89 min in room temperature group. Drawing on these statistics, a minimum of 24 patients were included in each group to detect a difference of onset time for 2 min between both groups with a 2-tailed α error = 0.05, and power 90%. Taking into account potential block failures and patient dropouts, 60 patients were enrolled.

### Statistical analysis

Data analysis was conducted using SPSS software version 27 (IBM Corporation, Armonk, NY). Normal distribution was evaluated using the Shapiro–Wilk test. Continuous variables for normality were expressed as mean ± SD and were analyzed with the Student t-test. Nonnormally distributed data was expressed as medians (interquartile range) and were analyzed with the Mann–Whitney U test. Categorical variables were presented as numbers (percentage). Data on ASA classification and gender were assessed using Chi-square. Fisher’s exact test was applied to text the differences in the side effects, patient satisfaction and types of operations between the two groups. A *P*-value < 0.05 was considered significant.

## Results

**Fig. 1:**
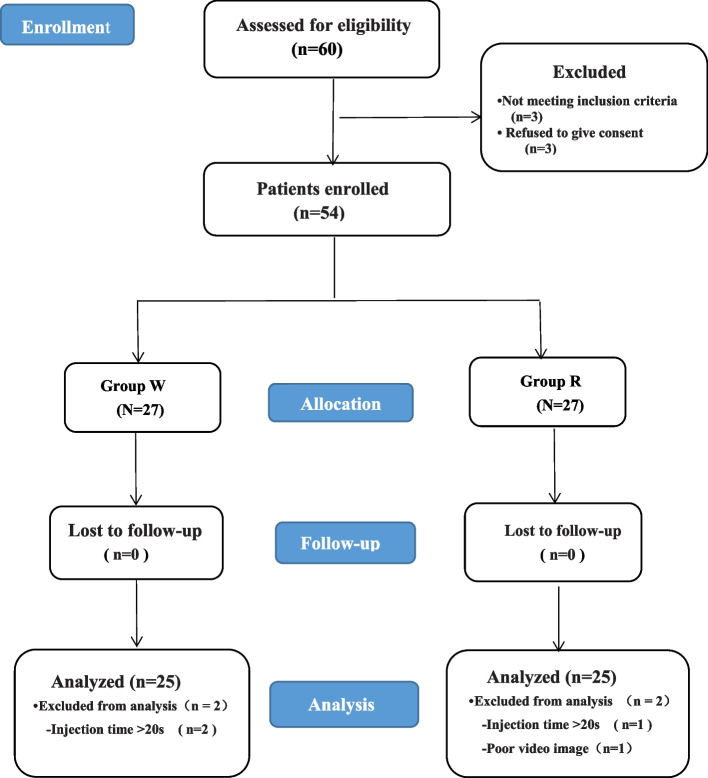
Patient flow diagram. Group W = ropivacaine warmed to 30 °C; Group R = ropivacaine at room tempeture (23 °C)

Sixty adult patients were evaluated for eligibility. However, three patients failed to fulfill the criteria for inclusion and three refused to participate. Therefore, 54 patients were ultimately enrolled, 27 in each group. Three patients (1 in group R and 2 in group W) were excluded for the injection time exceeding 20 s. In addition, one patient in group R was excluded on account of poor video image quality during the block. All the sciatic nerve blocks were successful and no patients were excluded due to inadequate block (Fig. 1). There were no significant differences in physical status, demographics, types of surgery, and the dimensions of the sciatic nerve between both gruops (Table 1).

**Table 1:** Patient demographics, surgical characteristics and sciatic nerve parameters

	**Group R (*n* = 25)**	**Group W (*n* = 25)**	**P**
**ASA, n (%)**			
**I**	**11 (44)**	**13 (52)**	**0.57**
**II**	**14 (56)**	**12 (48)**	
**Sex, n (%)**			
**F**	**12 (48)**	**12 (48)**	**1.0**
**M**	**13 (52)**	**13 (52)**	
**Age (years)**	**50.44 ± 11.17**	**51.12 ± 9.24**	**0.82**
**Weight (kg)**	**63.6 ± 11.82**	**64.16 ± 9.23**	**0.88**
**BMI** **(kg/m**^**2**^**)**	**23.63 ± 3.82**	**24.20 ± 2.54**	**0.52**
**Height (cm)**	**164.04 ± 9.65**	**162.48 ± 7.37**	**0.53**
**Sciatic nerve parameters (mm)**			
**Depth**	**29.32 ± 5.49**	**29.94 ± 4.92**	**0.68**
**Width**	**16.79 ± 1.58**	**16.35 ± 1.37**	**0.31**
**Thickness**	**4.77 ± 0.64**	**4.82 ± 0.72**	**0.79**
**Types of operation, n (%)**			
**Ankle**	**5 (20)**	**4 (16)**	**0.83**
**Metatarsal bones**	**4 (16)**	**2 (8)**	
**Calcaneus**	**2 (8)**	**2 ( 8)**	
**Tibiofibula**	**14 (56)**	**17 (68)**	

Values are presented as the mean ± SD or number of patients (percentage)

Group W = ropivacaine warmed to 30 °C

Group R = ropivacaine at room tempeture (23 °C)

*ASA* American Society of Anesthesiologists, *BMI* Body mass index

*p* < 0.05, statistically significant

The time to the onset of sensory blockade for each nerve in group W was shorter than in group R respectively: for the common peroneal nerve, (18 (14,18) min vs 22 (18,23) min, *P* < 0.001); for the sural nerve, (14 (12,16) min vs 20 (18,20)min, *P* < 0.001); for the tibial nerve, (12 (11,16) min vs 18 (16,20) min, *P* < 0.001). The time to the onset of limb sensory block was shorter in group W than in group R (16 (16,18) min vs 22 (20,23) min, *P* < 0.001) (Table 2).

**Table 2:** Onset and duration of sciatiac nerve blockade

	Group R (*n* = 25)	Group W (*n* = 25)	**P**
**Onset time of sensory block for each dermatome (min)**			
**In CPN distribution**	**22 (18,23)**	**18 (14,18)**	**< 0.001**
**In SN distribution**	**20 (18,20)**	**14 (12,16)**	**< 0.001**
**In TN distribution**	**18 (16,20)**	**12 (11,16)**	**< 0.001**
**Onset time of limb sensory block (min)**	**22 (20,23)**	**16 (16,18)**	**< 0.001**
**Onset time of motor block (min)**	**26 (24,28)**	**22 (20,24)**	**< 0.001**
**Analgesia duration (h)**	**14 (10.5,16)**	**13 (11,16)**	**0.62**
**Resolution of motor block (h)**	**12 (11,14)**	**12 (10,14.5)**	**0.68**

Data are presented as medians (interquartile ranges)

Group W = ropivacaine warmed to 30 °C; Group R = ropivacaine at room tempeture (23 °C)

*CPN* Common peroneal nerve, *TN* Tibial nerve, *SN* Sural nerve

*p* < 0.05, statistically significant

The time to the onset of motor blockade was shorter in group W than in group R (22 (20,24) min vs 26 (24,28) min, *P* < 0.001) (Table 2).

Duration of sensory blockade in group W and group R were 13 (11,16) minutes and 14 (10.5,16) minutes, respectively. Duration of motor blockade in group W and group R were 12 (10,14.5) minutes and 12 (11,14) minutes, respectively. There were no significant differences between both groups in the duration of sensory and motor blockade (*p* = 0.62 and *p* = 0.68) (Table 2).

The proportion of the patient satisfaction with the anesthetic effect was equal (92%vs 92%). There were no differences between both groups (*p* = 1) (Table 3).

**Table 3:** Neural complications and patient satisfaction

	Group R (*n* = 25)	Group W (*n* = 25)	**P**
**Neural complications, n (%)**			
**Paresthesia**	**0 (0)**	**0 (0)**	**1.0**
**Muscle weakness**	**0 (0)**	**0 (0)**	
**Patient satisfaction, n (%)**			
**Satisfaction**	**23 (92)**	**23 (92)**	**1.0**
**Dissatisfaction**	**2 (8)**	**2 (8)**	

Data are presented as number of patients (percentage)

Group W = ropivacaine warmed to 30 °C; Group R = Ropivacaine at room tempeture (23 °C)

*p* < 0.05, statistically significant

In both groups, adverse events such as intraneural injection and local anesthetic intoxication were not detected in all patients and no evident clinical neural symptoms were detected (*p* = 1) (Table 3).

## Discussion

In the present study, we demonstrated that warming ropivacaine 0.5% to 30℃ shortened the onset time of sensory and motor blockade in the ultrasound-guided subgluteal sciatic nerve block. The duration of sensory and motor blockade showed no significant difference between the studied groups. No evident clinical neural symptoms were detected and the proportion of the patient satisfaction with the anesthetic effect was equal in both groups.

The sciatic nerve is the largest peripheral nerve in the body whoes anatomical structure determines the prolonged latency of sciatic nerve block. The sciatic nerve tract is surrounded by a variety of non-neural tissues, primarily composed of abundant connective and adipose tissues, which serve as a physical barrier to slow down the diffusion of local anesthetics [2]. In addition, a significant amount of adipose tissues may serve as a reservoir for lipophilic local anesthetics, which reduce the quantity of local anesthetics diffusing into nerve tissues via the epineural membranes.

The basic mechanism that warming local anesthetics accelerate the onset of sensory and motor blockade still remains uncertain. The hypothetical explanation is that with temperature increasing, a lower pKa value of local anesthetic solutions leads to an increased proportion of non-ionized molecules which have greater lipophilicity to enhance the diffusion capacity of local anesthetics across tissues and nerve membranes [12, 13]. Additionally, on a thermodynamic basis, the increased thermal motion of local anesthetic molecules can partly accelerate the diffusion rate of local anesthetics [14]. However, we did not evaluate the pKa in this study. Maybe further measurement of the pKa will contribute to explaining our finding better in future study.

Similarly, in theory, the alkalinization of local anesthetics can also accelerate the onset time of nerve block by increasing the proportion of non-ionized molecules. However, the use of the alkalinization technique is limited in clinical practice for its uncertain efficacy, because the precipitation generated by alkalization of local anesthetic solutions may reduce drug bioavailability [4, 15]. The intraneural injection is an another safe and effective approach to influence the characters of the sciatic nerve block [16]. However, in terms of the onset time of the sciatic nerve block, the warming technique in our study seems to be superior.

Makharita et al. [17] reported that warming bupivacaine 0.5% from 23℃ to 37℃ reduced onset time of ultrasound-guided axillary plexus block by nearly 50%,while duration of sensory and motor blockade increased by nearly 40%. However, our results showed that the onset time of both the sensory and motor blockade decreased by about 22% and 16% respectively, but the difference of the duration of sensory and motor blockade between both groups was not significant when the ropivacaine was warmed from 23℃ to 30℃. We consider that apart from the differences in neural structure, temperature difference may also be an important factor resulting in the differences between the outcomes of the two studies. Therefore, We speculate that warming the ropivacaine to 37℃ will exert a more pronounced effect on the sciatic nerve block and it warrants further exploration.

Apart from peripheral nerve block, several studies have investigated the effect of warming local anesthetics on neuraxial block. Liu et al. [18] demonstrated that the administration of warming ropivacaine significantly accelerated the onset time of T12 and L3 sensory blockade for epidural anesthesia. Arai et al. [19] discovered that warming caused a reduction in the viscosity of bupivacaine solutions, resulting in an elevation of the cephalad level of spinal anesthesia. Another study [20] showed an interesting phenomenon that warming the heavy bupivacaine to 37 °C could decrease the occurrence and severity of shivering and pruritis secondary in parturient candidates undergoing cesarean sections with spinal anesthesia.

In an animal study, Nikolaos et al. [21] compared the influence of lidocaine with different temperatures on the sciatic nerve of the rats in vitro and in vivo through electrophysiological recordings. The finding indicated that lidocaine administered at 4℃ exhibited greater potency with respect to the establishment and duration of anesthesia, as opposed to 25℃. However, it was contrary to our finding in this study. Maybe the anatomical dissimilarities of the sciatic nerve between human and rat accounted for this difference between the outcomes of two studies. Another study [9] showed that the use of the local anesthetics at lower temperatures may lead to a delayed onset of both motor and sensory blockade in the infraclavicular brachial plexus nerve block. Therefore, a further investigation is warranted to investigate the impact of local anesthetics at low temperatures on sciatic nerve blockade.

We recognize several limitations in our study. First, we just warmed the ropivacaine to 30℃, which was different from previous studies where the target temperature was set at 37℃. Since the storage temperature of ropivacaine should be strictly controlled below 30℃ as specified, the off-label use was not permitted by the ethics committee for safety reasons. Currently, the effect of warming temperature beyond 30℃ on the ingredients, properties and potential cytotoxicity [22] of ropivacaine are unavailable and it warrants further exploration. Second, we did not assess the plasma bupivacaine levels. In theory, warming temperature may enhance blood circulation of surrounding tissues, resulting in increased absorption of ropivacaine into the plasma, which may increase the risk of systemic toxicity. Thus the evaluation of the systemic absorption of local anaesthetics is necessary in future study. Third, we evaluated the complications including dysesthesia, paresthesia, and/or motor weakness just 2 days after surgery. Maybe a longer follow-up will be needed to observe the long-term complications. Although there are no evident clinical neural symptoms in our study, the application of the electrophysiologic tests and the evaluation of the nerve histologic changes are still useful to assess the potential neurological damage at various warming temperatures in future study. Finally, our study had a small sample size in a single center and this small sample size may not be representative of the broader patient population. In addition, various exclusion criterias (e.g. injection time exceeding 20 s) were used to ensure accurate experimental results, however, they may introduce selection bias. Thus, further multicenter studies with a larger and more diverse sample are needed to endorse the results of this study.

## Conclusions

In conclusion, our study shows that warming the ropivacaine 0.5% to 30℃ can accelerate the onset time of the ultrasound-guided subgluteal sciatic nerve block whithout obvious adverse effects. Therefore, warming the local anesthetics is a simlpe, safe and effective method to reduce the latency of peripheral nerve block, which is of benefit to shorten patient waiting time in the preoperative room and improve the clinical efficiency in busy surgical settings.

## Data Availability

The data associated with the paper are not publicly available but are available from the corresponding author (YG) on reasonable request.

## Abbreviations

ASA: The American Society of Anesthesiologists
BP: Blood pressure
HR: Heart rate
ECG: Electrocardiograph
SpO2: Pulse oxygen saturation
CPN: Common peroneal nerve
TN: Tibial nerve
SN: Sural nerve
